# A randomised controlled trial of time limited CBT informed psychological therapy for anxiety in bipolar disorder

**DOI:** 10.1186/1471-244X-13-54

**Published:** 2013-02-15

**Authors:** Steven Jones, Elly McGrath, Kay Hampshire, Rebecca Owen, Lisa Riste, Chris Roberts, Linda Davies, Debbie Mayes

**Affiliations:** 1 Spectrum Centre for Mental Health Research, School of Health and Medicine, Lancaster University, Lancaster, UK; 2 Spectrum Centre for Mental Health Research, School of Health and Medicine, Lancaster University & Manchester Mental Health and Social Care Trust, Lancaster, UK; 3 Manchester Mental Health and Social Care Trust, Manchester, UK; 4 Manchester Mental Health and Social Care Trust and University of Manchester, Manchester, UK; 5 School of Medicine, University of Manchester, Manchester, UK

## Abstract

**Background:**

Anxiety comorbidity is common in bipolar disorder and is associated with worse treatment outcomes, greater risk of self harm, suicide and substance misuse. To date however there have been no psychological interventions specifically designed to address this problem. The primary objective of this trial is to establish the acceptability and feasibility of a new integrated intervention for anxiety in bipolar disorder designed in collaboration with individuals with personal experience of both problems.

**Methods and design:**

Single blind randomised controlled trials to assess the feasibility and acceptability of a time limited CBT informed psychological intervention for anxiety in bipolar disorder (AIBD) compared with treatment as usual. Participants will be recruited from across the North West of England from specialist mental health services and through primary care and self referral. The primary outcome of the study is the feasibility and acceptability of AIBD assessed by recruitment to target and retention to follow-up, as well as absence of untoward incidents associated with AIBD. We will also estimate the effect size of the impact of the intervention on anxiety and mood outcomes, as well as calculate preliminary estimates of cost-effectiveness and investigate potential mechanisms for this (stigma, self appraisal and stability of social rhythms).

**Discussion:**

This is the first trial of an integrated intervention for anxiety in bipolar disorder. It is of interest to researchers involved in the development of new therapies for bipolar disorder as well as indicating the wider potential for evaluating approaches to the treatment of comorbidity in severe mental illness.

**Trial registration number:**

ISRCTN84288072

## Background

Bipolar disorder is a serious public health problem which affects 1-2% of the UK population. It is characterised by periods of high and low mood (depression and mania) and has potentially lifelong impact on the individual, carers and wider society [1,2]. It is the sixth leading cause of disability among people aged 15–44 years [3] and costs the UK £2 billion per annum [4]. Provision of psychological treatment is a key NICE recommendation for BD and is cost effective by preventing hospitalisation [1,5].

Psychological treatment to delay the onset or minimise the severity of episodes and optimise function is efficacious, cost effective and popular with service users [1] but none of the established treatments are designed to specifically address anxiety, despite strong evidence for the effectiveness of psychological interventions for anxiety not specific to bipolar disorder [6]. This is even more of a concern as anxiety is highly comorbid (93% lifetime [7] and 32% current comorbid anxiety [8]) with bipolar disorder. Comorbid anxiety is an important issue in bipolar disorder due to the distress it causes patients and specifically due to the negative impact it can have on the course and outcome of the condition. Challenges asssociated with anxiety cormorbidity in bipolar disorder include; poor treatment response [9], increases in suicidality [10], earlier age of onset of BD [11], which is also associated with higher levels of suicidality [12], and greater relapse risk [8]. Effective time-limited interventions for anxiety already exist and would therefore have the potential to improve a range of adverse outcomes in BD.

Provencher and colleagues [13] recently reviewed treatment studies that included individuals with bipolar disorder and anxiety (either symptomatic or as specific anxiety diagnosis). They reported promising results, a number of small scale single case and case series studies of psychological treatment of anxiety in bipolar disorder producing a reduction of anxiety symptoms. However, they also highlighted the absence of randomised controlled trials specifically designed to evaluate an integrated approach to the treatment of anxiety in bipolar disorder. A key question in developing approaches to anxiety comorbidity is whether to target specific anxiety diagnostic categories or anxiety symptoms. The latter course was chosen for this study for several reasons. Firstly, anxiety disorders themselves tend to be highly comorbid especially in bipolar disorder [13], suggesting a risk that large numbers of parallel interventions might be required making the therapy unwieldy to develop, deliver and assess. Secondly, there is evidence from our own team and other researchers that a substantial proportion of individuals with bipolar disorder experience significant distress associated with anxiety without fitting neatly within a single anxiety diagnostic category. Thirdly, although the detailed manifestations of anxiety disorders differ, they typically share key elements in common including subjective feelings of anxiety, worry and tension and interference with functioning. Fourthly, our findings from interviews and focus groups with service users and the advice of our service user reference group was that this approach would open the trial to the widest possible range of individuals with bipolar disorder who might potentially benefit from it. Consistent with their proposal that there is an urgent need for studies of this type, the present study is an RCT feasability study of a new psychological treatment for the reduction of anxiety in bipolar disorder.

This study constitutes the third and final phase in a treatment development study. The preceding phases in preparation for this study were: i) qualitative interviews conducted with bipolar individuals concerning their experiences of anxiety and their views about psychological treatment; and ii) a series of focus groups conducted with services users and health professionals to develop and refine the treatment manual employed here. The findings of both previous phases have informed the content and presentation of the therapy evaluated here, consistent with wider recogntion of the crucial importance of involving service users in treatment development, planning and provision [14]. There is an increasing recognition that qualitative methods are better suited to understanding the perspectives of service users and capitalizing on their insights [15]. The present study draws on qualitative methods in the earlier phases as a foundation to understand the service user perspective from which the intervention being tested has been developed, alongside the current evidence base. Provencher has argued that the strongest evidence to date is for the benefits of cognitive behavioural treatments in addressing anxiety in bipolar disorder without negatively impacting on affective symptoms. The present therapeutic approach is based on an integration of structured psychological therapy for bipolar disorder with effective anxiety therapies as identified in the NICE bipolar and anxiety guidelines respectively [1,16]. In this study we evaluate the feasibility and acceptability of delivering an integrated time limited anxiety intervention to individuals with bipolar disorder (AIBD). As a feasibility study we primarily evaluate recruitment into the study and consent to participate, adherence to the intervention, retention within both arms across assessment, intervention and follow-up periods and outcome parameter estimates. From this we will be able to assess the acceptability of the intervention to service users. In addition, this trial will also provide initial evidence of the clinical impact of the intervention with respect to anxiety, mood symptoms and relapse as potential primary outcomes for a definitive clinical randomised controlled trial in the future.

## Method

This RCT is conducted by a multidisciplinary team of researchers, clinicians, statisticians and therapists across academic institutions and NHS Trusts in the North West of England. This study was reviewed and approved by the UK NHS Ethics Committee process (REC ref: 10/H1015/83).

### Objective

To determine the feasibility and acceptability of an integrated cognitive behaviour therapy intervention for anxiety in the context of bipolar disorder compared with treatment as usual.

Main research questions:

• To demonstrate feasibility of recruitment and consenting procedures, adherence to protocol and retention to both arms of the trial across assessment, intervention and 4 month follow-up periods extending up to 20 months post randomisation.

• To provide parameter estimates of clinical outcomes with respect to bipolar relapse, mood and anxiety symptoms, suicidality, recovery, cognitive style, quality of life and cost effectiveness.

### Trial design

A rater-blind randomised controlled trial which compares: i) 10 sessions of integrated CBT for anxiety in the context of bipolar disorder with; ii) treatment as usual. The trial is based in the North West of England with recruitment across this region sampling individuals across rural and urban areas and a wide range of settings with respect to sociodemographic status and ethnic mix.

Randomisation is carried out using randomly sized permuted blocks, by the independent Clinical Trials Unit at The Christie NHS Foundation Trust, Manchester. Minimisation is used with respect to gender, number of previous bipolar episodes (mania, hypomania or depression) and level of current anxiety. These were selected as there is evidence that clinical outcomes from psychological therapy are typically better for females [17] and for those with lower anxiety [13] and to a lesser extent there is evidence for better outcomes in bipolar disorder for individuals with fewer previous episodes [18,19].

### Sample

#### Sample size

As the primary purpose of the study is to evaluate the feasibility and acceptability of delivering the proposed intervention a formal power calculation is not appropriate. It has been estimated that 30 participants per group will be sufficient to be able to reliably determine primary feasibility outcomes. The recruitment target is set at 72 participants to allow for expected attrition rates and measures of clinical outcome will be recorded at baseline and follow-up to provide an indication of the effectiveness of the intervention in decreasing anxiety, regulating mood and its impact on other clinical outcomes. This number will also allow us to evaluate the secondary objective of the trial; to estimate the potential treatment effect size as the basis and justification for a further, more definitive trial.

#### Recruitment

**Figure 1:**
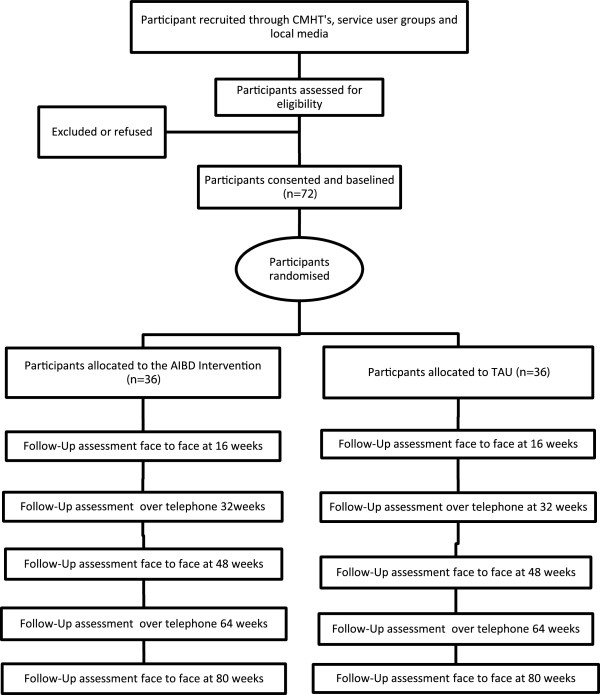
Diagram showing the design of the study.

Seven NHS Trusts in the North West UK are taking part in this study; Manchester Mental Health and Social Care Trust, Lancashire Care NHS Foundation Trust, North Lancashire Primary Care Trust, Cumbria Partnership NHS Foundation Trust, Cumbria Primary Care Trust, Blackpool Primary Care Trust and Merseycare NHS Trust. Community mental health teams, out-patient clinics, GP surgeries, primary care mental health teams and voluntary services are approached to identify potential participants. Care co-ordinators, research nurses and research development officers are encouraged to contact potential participants to introduce the trial. When recruiting in community mental health teams and voluntary services, a member of the research team presents an outline of the study and provides written material about it. Potential participants are given a participant information sheet by their care co-ordinators or the research team, outlining the study and their role should they wish to take part. The study is also advertised in local media and posters and leaflets distributed in both NHS and non NHS sites to maximise participant access. Care co-ordinators and other relevant health professionals are informed of a participant’s involvement in the study with the participant’s consent. If a participant does not wish their GP or care coordinator to be informed about their involvement in the study, this does not prevent their participation although contact details of all health professionals involved in their care are still required in the case of any clinical adverse events during the study. Participants are made aware on entry to the study that their care co-ordinator will be contacted should they be a significant risk to themselves or others during the study. Figure 1 gives an outline of the design of the study.

#### Inclusion/exclusion criteria

Potential participants must meet the inclusion criteria of:

• SCID DSM-IV diagnosis of primary bipolar disorder [20],

• Current HADS-A score of 8 or more [21],

• Sufficient understanding of written and spoken English to engage with interviews and use the intervention material.

• Aged 18 or over.

Exclusion criteria

• Manic, hypomanic, depressed or mixed episode currently or in the last four weeks

• Current suicidal ideation with intent

• Unable or unwilling to provide informed consent

### Outcome measures

In order to evaluate the feasibility and acceptability of delivering this time limited anxiety intervention to individuals with a diagnosis of bipolar disorder the following data will be evaluated:

Levels of recruitment into the trial, retention of participants in both arms of the study and adherence to and completion of the intervention. At baseline the Structured Clinical Interview for DSM-IV is completed to confirm bipolar diagnosis and to provide information on anxiety disorder diagnoses as well as information on sociodemographic variables. Measures of clinical outcome will be recorded at baseline and follow-ups to provide an indication of the effectiveness of the intervention in decreasing anxiety, regulating mood and its impact on other clinical outcomes.

#### Primary clinical outcomes

Hypotheses for primary clinical outcomes are that AIBD will i) reduce anxiety symptoms assessed by means of the observer rated *Hamilton Anxiety and Depression Rating Scale* (HAM-AD) [22] and by the self report *State*-*Trait Anxiety Inventory for Adults* (STAI) [23]; ii) increase time to relapses of mood episodes as measured by *Structured Clinical Interview for Diagnosis*: *Research Version* (SCID DSM-IV: SCID Life) [20]; and iii) reduce mood symptoms as measured by *Hamilton Anxiety and Depression Rating Scale* (HAM-AD) [22] and *Bech*-*Rafaelsen Mania Scale* (MAS) [24].

#### Secondary clinical outcome measures

Hypotheses for secondary outcomes are that AIBD will improve i) Quality of life and social functioning as measured by *EuroQol* scale (EQ-5D) [25], *Quality of Life in Bipolar Disorder Questionnaire* (QoL.BD) [26], and the observer rated *Personal and Social Performance Scale* (PSP) [27]; ii) Recovery as measured by the *Bipolar Recovery Questionnaire* (BRQ ) [28]; iii) Medication adherence measured by *Stephenson Medical Adherence Questionnaire* (MEDAD) [29]. It is also hypothesised that AIBD will cost no more than TAU as assessed by *Client Service Receipt Inventory* (CSRI) [30], the *Economic Patient Questionnaire* (EPQ) (Davies, pers comm.) and informed by EQ-5D as a measure of health status.

#### Process measures

Hypotheses for process measures are that AIBD will improve clinical outcomes through; i) reducing tendency towards positive self appraisals as measured by the *Hypomanic Interpretations Questionnaire* (HIQ) [31]; ii) Stabilising activity and sleep patterns as measured by *The Social Rhythm Metric* (SRM-T) [32]; and iii) reducing experiences of stigma measured by the *Hayward Stigma Questionnaire* - *Revised Version* (HSQ) [33] – self-report questionnaire measuring the experiences of stigma.

#### Measures to assess therapeutic alliance and adherence to treatment protocol

Engagement in therapy will be assessed by means of the *Work Alliance Inventory* (short-form, therapist and client versions: WAI-S) [34]. Treatment fidelity will be assessed by both the Cognitive Therapy Scale Revised version and the AIBD Fidelity Scale specifically designed for the current study.

Participants are assessed on all measures at baseline, no more than 2 weeks prior to randomisation and, where applicable, the start of therapy and at 4, 12 and 20 month follow-up appointments administered in person. A sub-set of follow-up measures (SCID life, including the MAS, the HAM-D and the HAM-A, and the MEDAD) are also to be carried out by telephone at 8 and 16 months. Table 1 shows the timeframe for the measures. Assessments are recorded with consent from the participant (Table 1).

**Table 1:** Schedule of quantitative assessments for service users

**Assessment**	**Initial interview**	**Baseline**	**12 weeks**	**36 weeks (telephone)**	**48 weeks**	**60 weeks (telephone)**	**72 weeks**
			Primary Outcome Measures				
Hamilton Anxiety and Depression Rating Scale (Ham-AD)	-	+	+	+	+	+	+
State-Trait Anxiety Inventory for Adults (STAI)	-	+	+	-	+	+	+
Structured Clinical Interview for Diagnosis (SCID-IV)	+	-	-	-	-	-	-
SCID-Life	-	+	+	+	+	+	+
Bech-Rafaelsen Mania Scale (MAS)	-	+	+	+	+	+	+
			Secondary Outcome Measures				
Euroquol Scale (EQ5D)	-	+	+	-	+	-	+
Quality of Life in Bipolar Disorder Questionnaire (QOL-BD)	-	+	+	-	+	-	+
Personal and Social Performance Scale (PSP)	-	+	+	-	+	-	+
Bipolar Recovery Questionnaire BRQ	-	+	+	-	+	-	+
Stephenson Medication Adherence (MED-AD)	-	+	+	+	+	+	+
Economic Patient Questionnaire (EPQ)	-	+	+	-	+	-	+
Client Service Receipt Inventory (CSRI)	-	+	+	-	+	-	+
			Process Measures				
Hypomanic Interpretations Questionnaire (HIQ)	-	+	+	-	+	-	+
Social Rhythm Metric (SRM-T)	-	+	+	-	+	-	+
Hayward Stigma Questionnaire (HSQ)	-	+	+	-	+	-	+
			Potential Confounds				
Demographics	+	-	-	-	-	-	-
Clinical Data*	+	+	+	+	+	+	+

Note: + indicates that the assessment will be used at the specified interview and - indicates that it will not.

*Clinical Data includes start and end date of last episode and total number of episodes.

### The AIBD intervention

#### Development

The intervention combines current knowledge and evidence for the effectiveness of CBT in reducing anxiety with data collected in phases 1 and 2 of this study in collaboration with individuals with a diagnosis of bipolar disorder. In line with suggestions that it would be useful to adapt the CBT protocol to include modules based on a client’s clinical anxiety profile [8], this has been done with the current intervention. The intervention takes into account the views from service users who participated in the focus groups and individual interviews of the first two phases of this study, as well as explicitly recommended by the National Institute for Health and Clinical Excellence (NICE) guidelines for bipolar disorder and anxiety. The importance of the therapeutic alliance and the flexibility of appointments and trustworthiness of the therapist were highlighted as paramount during the individual interviews and later validated by the focus groups, and so were included. The intervention includes the elements recommended by the NICE guidelines as being useful in addressing anxiety with CBT, namely i) improving awareness of symptoms, ii) mental imagery [35], iii) relaxation techniques and iv) gradual exposure to, and mastery of, anxiety provoking situation [36]. These in turn reflect the methods used in previous studies approved by NICE guidelines [37,38]. As the intervention is aimed at reducing anxiety in those with bipolar disorder, the intervention pays particular attention to the dynamic interplay between anxiety and mood instability. By drawing on existing evidence, the therapist will also work with the service user to improve stability of mood by using mood monitoring techniques, regularisation of routine and improving problem solving techniques where appropriate. The exact balance of therapy approaches is determined individually based on client's primary therapy goals and a formulation of their anxiety issues in relation to bipolar disorder. Other important considerations emerging from the service user perspective are the need for more information about bipolar disorder in general and the need for an awareness of different levels of anxiety and the subsequent ability to implement new skills learnt in therapy. As a result, and also in line with research evidence that self-help material contributes towards better clinical outcome [39], participants are provided with both service user manuals and access to a web page to provide personal access to all information used in therapy online.

### Analysis

#### Feasibility

As the primary purpose of the study is to evaluate the feasibility and acceptability of delivering the proposed intervention a formal power calculation comparing treatment groups is not essential. With 72 subjects in total the study will estimate a follow-up rate of 75% with precision +/- 10%.

#### Clinical outcomes

Quantitative outcomes (primary, secondary and process) will be analysed using a linear model analysis of covariance adjusting for baseline values of the outcome measure as well as gender, current anxiety and number of prior episode as pre-specified in the statistical analysis plan. Ordered categorical outcomes will be analysed using ordinal logistic regression which estimates the odds of a participant being in a higher category in the intervention compared to the control.

A longitudinal analysis will also be carried out using a linear and ordinal logistic regression mixed model including all data from follow-up time points with baseline values as a covariate so as to maximize the power to detect differences between treatment groups.

#### Cost effectiveness

The EQ-5D scores at each assessment will be converted into a single index value, using published utility weights. These will be used to estimate quality adjusted life years (QALYs), which will be the outcome measure for the economic analysis [40].

Descriptive and regression analyses will be used to identify key elements of service use and cost to inform the design of data collection for the economic evaluation integrated into the future clinical trial. Descriptive and regression analysis will also be used to explore the potential impact of baseline participant and service characteristics on the costs and QALYs. This will be used to identify potential covariates that may be important to include in the analysis of cost effectiveness. The analysis will also be used to identify possible mediators that may influence the cost effectiveness of therapy. The economic analysis will estimate the costs of health and social care and quality adjusted life years (QALYs) from a broadly societal perspective. The primary and secondary economic analyses, controlled for confounds, will estimate incremental cost effectiveness ratios, cost effectiveness acceptability curves and net benefit statistics of CBT compared to TAU. This uses a Bayesian approach to estimate the likelihood that CBT is cost effective without hypothesis testing and risk of a Type II error. Secondary and sensitivity analysis will be used to further assess the robustness of the results.

#### Effect size

A significance level of 25% will be used instead of the usual 5% in this circumstance as the current study is a pilot study [41]. With 36 participants in each treatment group, the study would have 75% power to detect a standardised effect size of 0.5 across anxiety symptoms as measured using the HAM-A, matching related studies [18,42-44], assuming 75% follow-up and a 25% two-sided significance level. Power will be increased by adjustment for baseline values of outcome measure in the statistical analysis using a linear model.

## Discussion

This study will provide feasibility information and preliminary effect size estimates to inform the development and evaluation of a definitive CBT intervention for anxiety in bipolar disorder. The AIBD intervention was developed in partnership with individuals with lived experience of bipolar consistent with UK Mental Health Research Network Good Practice Guidelines [45], including service user oversight of preparatory qualitative work on experiences of anxiety and wishes for anxiety therapy in bipolar disorder and the structure and format of the therapy. This level of engagement of individuals is also consistent with wider research on the importance of collaborative approaches to improving outcomes for clinical interventions [46-48]. AIBD is also informed by evidence based components of effective therapy for anxiety and bipolar disorder derived from the respective guidelines [1,6]. AIBD has a strong emphasis on formulation permitting client and therapist to negotiate the relative balance of anxiety and bipolar specific therapy elements on an individual basis.

Strengths of the study include targeting a clearly defined sample who to date have received little help with their anxiety problems and expressed great enthusiasm for the type of treatment described here in both qualitative and focus group works preparatory to this trial. By recruiting from across NHS primary and secondary care settings and through self referral findings should be more representative than those that solely focus on specialist mental health settings, as only a subset of individuals with bipolar disorder are in such settings long term [49].

There are weaknesses to the study which would need to be addressed in a definitive trial. Firstly, there is no active treatment control group so that any indications of effectiveness need to be interpreted with caution as we will not know whether possible benefits are a function of this specific treatment or structured treatment in general. Secondly, the scale of the study allows us to follow participants for up to 12 months following therapy completion. Longer follow-ups would be helpful to indicate more definitively whether this intervention impacts on relapse and the duration of impact on anxiety. Thirdly, the very individualised nature of the intervention means that it was necessary to devise a new measure specifically to explore fidelity to a very individualised therapy protocol. As we are uncertain how this will perform we have also used the established CTS-R which might underestimate fidelity in a flexible therapy of this type.

Despite these challenges, if the current study indicates that AIBD is feasible and has potential clinical benefits it will be an important step towards developing integrated approaches to anxiety comorbidity interventions for people with bipolar disorder that have been lacking until now.

## Competing interests

The authors declare that they have no competing interests.

## Authors’ contributions

SJ is the Chief Investigator for the PARADES Programme, principal investigator for this study, responsible to the conduct of the study and wrote the first draft of the paper. EM and KH contributed to recruitment and follow-up of study participants and contributed to protocol design and write up of the current paper. RO & LR co-ordinated the study in the context of the wider PARADES Programme. CR is the trial statistician and is a grant holder. LD leads the economic analysis and is a grant holder. DM leads the analysis from a service user perspective and is a grant holder. All authors contributed to the design of the study, revised the manuscript and gave final approval to the manuscript.

## Funding

National Institute for Health Research, England.

Programme Grant for Applied Research (PGFAR).

## Pre-publication history

The pre-publication history for this paper can be accessed here:

http://www.biomedcentral.com/1471-244X/13/54/prepub
